# Supplementary material for: Ultrarare Loss-of-Function Mutations in the Genes Encoding the Ionotropic Glutamate Receptors of Kainate Subtypes Associated with Schizophrenia Disrupt the Interaction with PSD95
Source: J Pers Med. 2022 May 12;12(5):783. doi: 10.3390/jpm12050783 (PMC9144110; doi:10.3390/jpm12050783)
Supplement: Supplementary file 1 [file jpm-12-00783-s001.zip › Supplementary Figures 1.pdf]

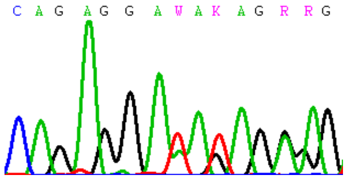

GRIK1:c.70_71delTT (p.Phe24fs)

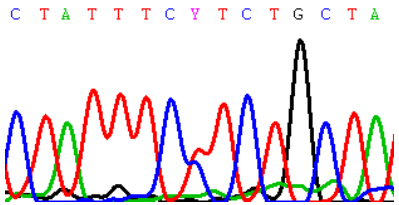

GRIK1:c.73C>T (p.Leu25Phe)

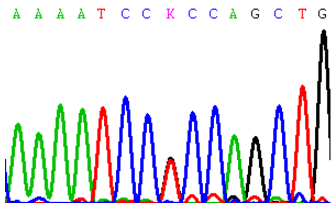

GRIK1:c.608G>T (p.Arg203Leu)

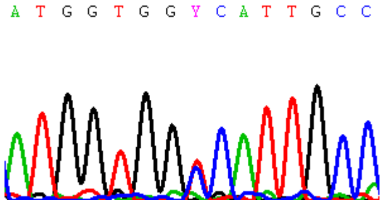

GRIK1:c.995C>T (p.Ala332Val)

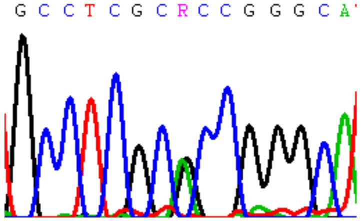

GRIK1:c.1007A>G (p.His336Arg)

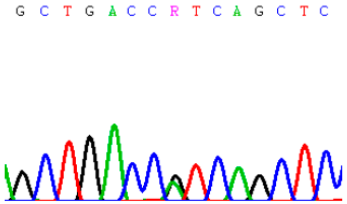

GRIK1:c.1027G>A (p.Val343Ile)

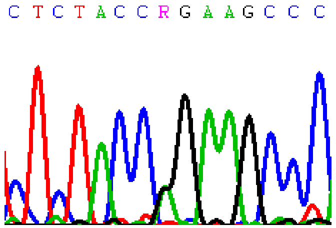

GRIK1:c.1673G>A (p.Arg558Gln)

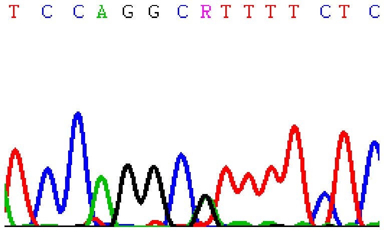

GRIK1:c.1699G>A (p.Val567Ile)

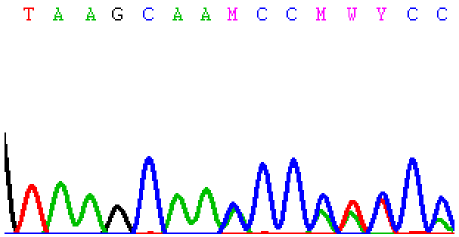

GRIK1:c.2644delA (p.Thr882fs)

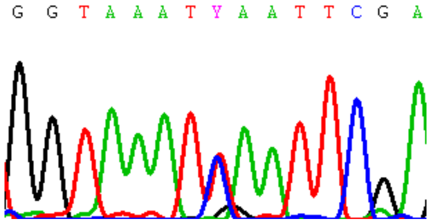

GRIK1:c.2705T>C (p.Leu902Ser)

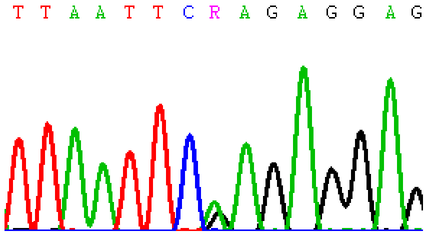

GRIK1:c.2711G>A (p.Arg904Gln)

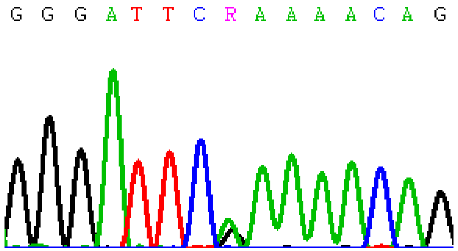

GRIK1:c.2729G>A (p.Arg910Gln)

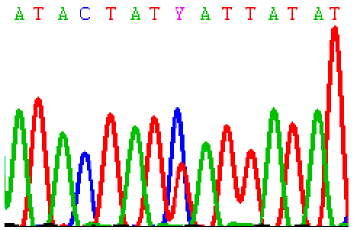

GRIK2:c.757C>T (p.His253Tyr)

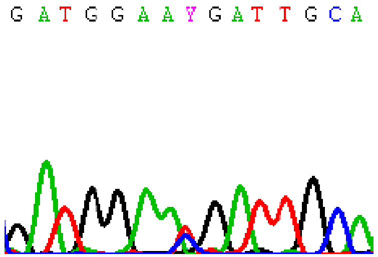

GRIK2:c.898C>T (p.Arg300Ter)

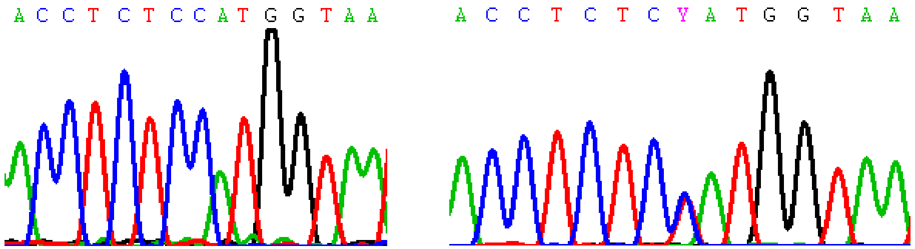

GRIK2:c.1360T>C (p.Tyr454His)

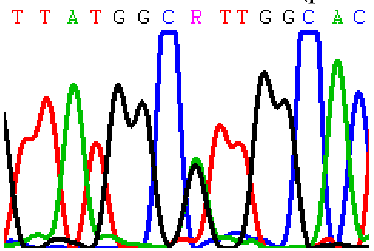

GRIK2:c.2296G>A (p.Val766Ile)

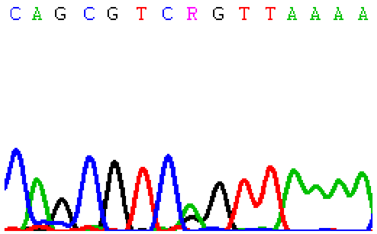

GRIK2:c.2621G>A (p.Arg874Gln)

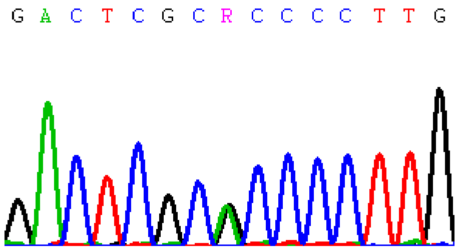

GRIK3:c.644G>A (p.Arg215His)

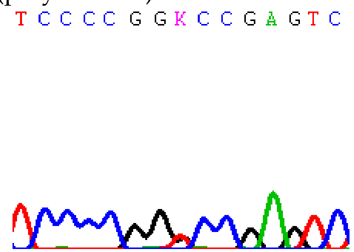

GRIK3:c.928T>G (p.Ser310Ala)

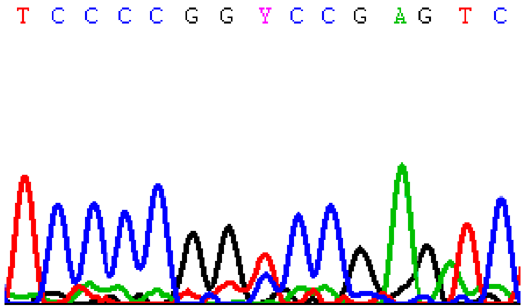

GRIK3:c.928T>C (p.Ser310Pro)

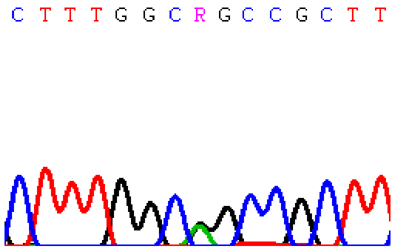

GRIK3:c.1078G>A (p.Gly360Ser)

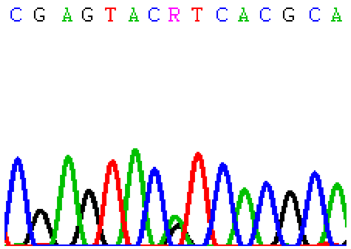

GRIK3:c.2236G>A (p.Val746Ile)

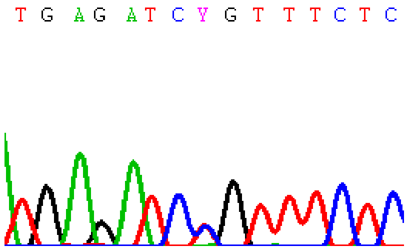

GRIK3:c.2599C>T (.Arg867Cys)

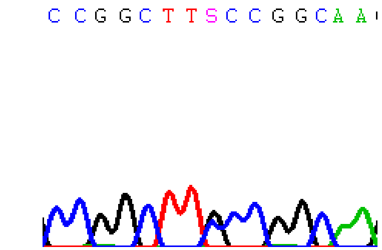

GRIK3:c.2707C>G (p.Pro903Ala)

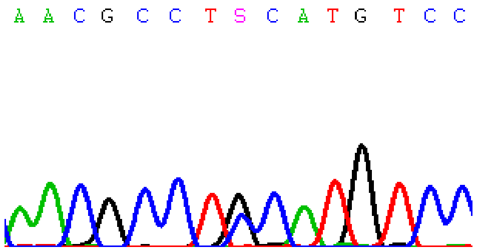

GRIK4:c.665C>G (p.Ser222Cys)

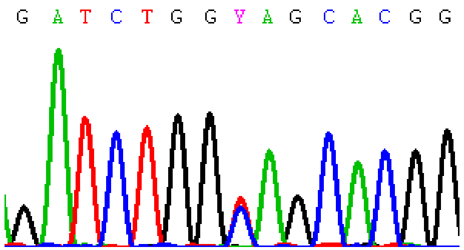

GRIK4:c.1024C>T (p.Gln342Ter)

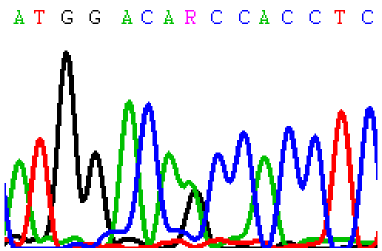

GRIK4:c.1205G>A (p.Ser402Asn)

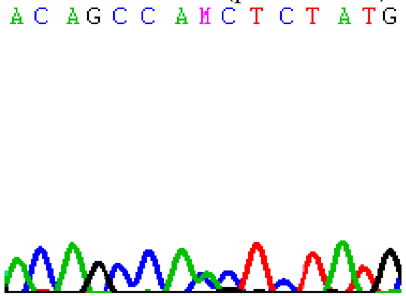

GRIK4:c.1209C>A (p.His403Gln)

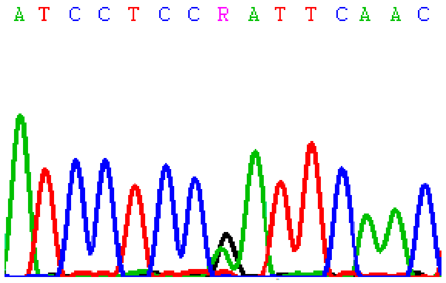

GRIK4:c.1379G>A (p.Arg460Gln)

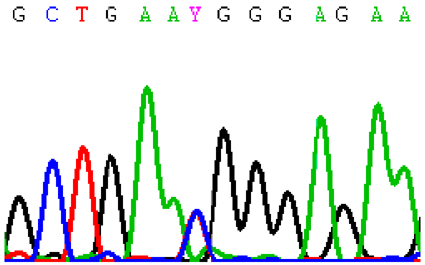

GRIK4:c.1519C>T (p.Arg507Trp)

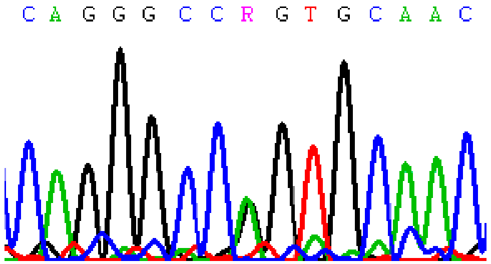

GRIK4:c.1748G>A (p.Arg583Gln)

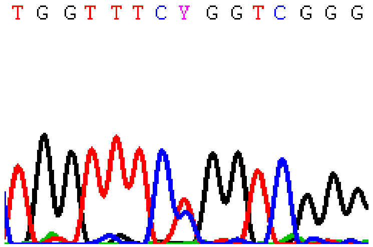

GRIK4:c.1799C>T (p.Pro600Leu)

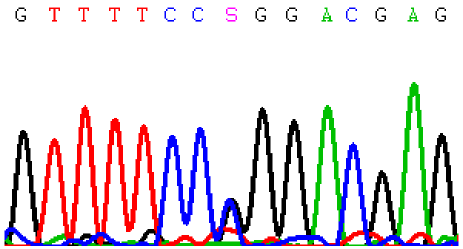

GRIK4:c.2279G>C (p.Arg760Pro)

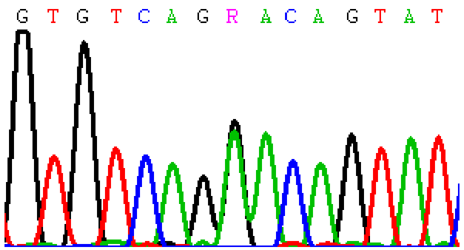

GRIK4:c.2569G>A (p.Asp857Asn)

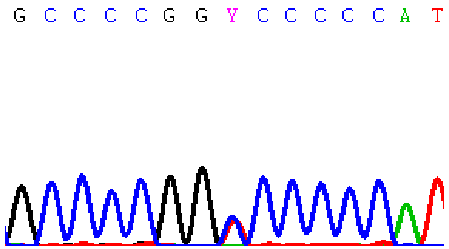

GRIK4:c.2617C>T (p.Pro873Ser)

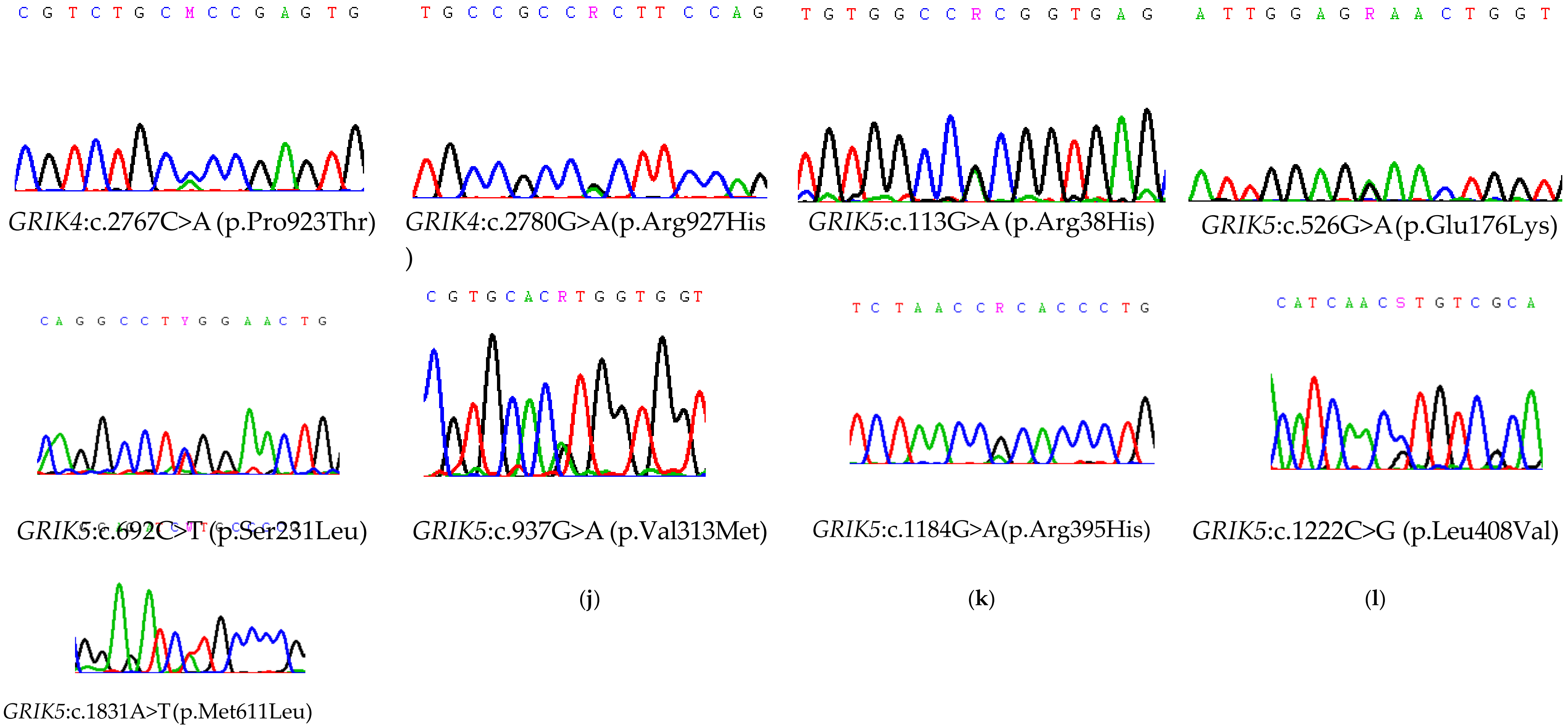

Supplementary Figure S1. Sequence electropherograms of the protein-altering variants of the GRIK gene family found in patients with schizophrenia.
