# Supplementary material for: Ultrarare Loss-of-Function Mutations in the Genes Encoding the Ionotropic Glutamate Receptors of Kainate Subtypes Associated with Schizophrenia Disrupt the Interaction with PSD95
Source: J Pers Med. 2022 May 12;12(5):783. doi: 10.3390/jpm12050783 (PMC9144110; doi:10.3390/jpm12050783)
Supplement: Supplementary file 1 [file jpm-12-00783-s001.zip › Supplementary Figures 2-3.pdf]

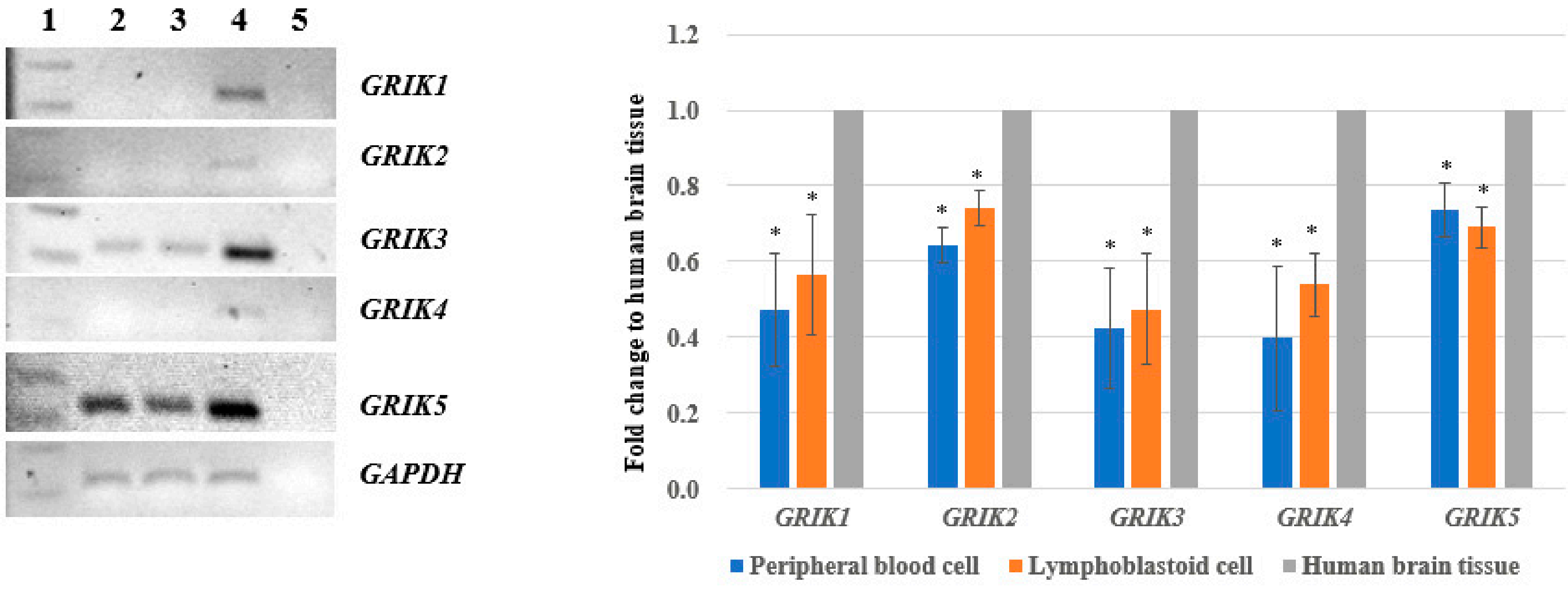

Supplementary Figure S2. The mRNA expression of *GRIK1*, *GRIK2*, *GRIK3*, *GRIK4*, and *GRIK5* genes in peripheral blood cells, lymphoblastoid cells, and the human brain. Lane 1 is the 100 bp ladder DNA marker; lane 2 is the peripheral blood cell; lane 3 is the lymphoblastoid cell; lane 4 is the human brain tissue; lane 5 is the negative control. The *GAPDH* gene was used as an internal control gene. The differences in gene expression activity between the two groups (compared with human brain tissue) were analyzed using Student's t-tests. (* $p < 0.05$; $n = 3$)

(A)

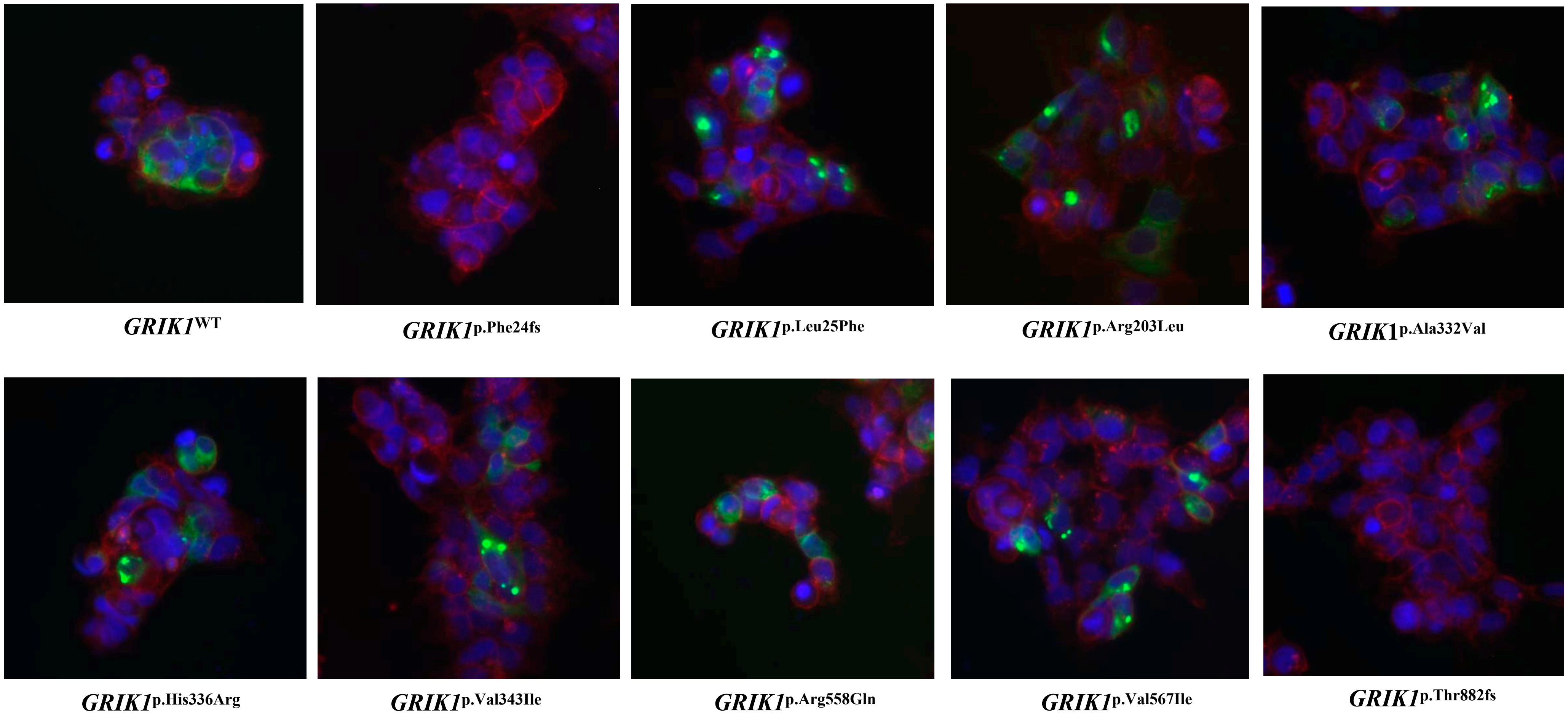
GRIK1WT
GRIK1p.Phe24fs
GRIK1p.Leu25Phe
GRIK1p.Arg203Leu
GRIK1p.Ala332Val
GRIK1p.His336Arg
GRIK1p.Val343Ile
GRIK1p.Arg558Gln
GRIK1p.Val567Ile
GRIK1p.Thr882fs

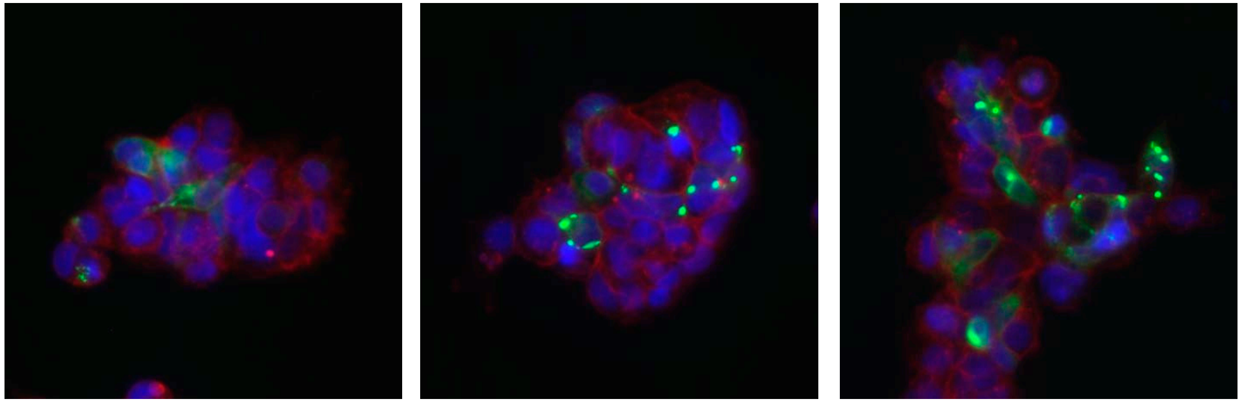

*GRIK1*p.Leu902Ser *GRIK1*p.Arg904Gln *GRIK1*p.Arg910Gln

(B)

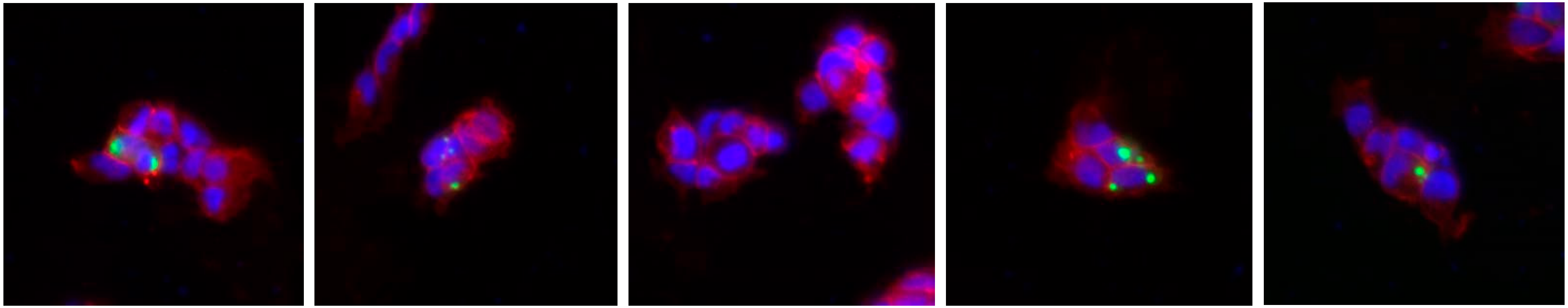

*GRIK2*WT *GRIK2*p.His253Tyr *GRIK2*p.Arg300Ter *GRIK2*p.Tyr454His *GRIK2*p.Val766Ile

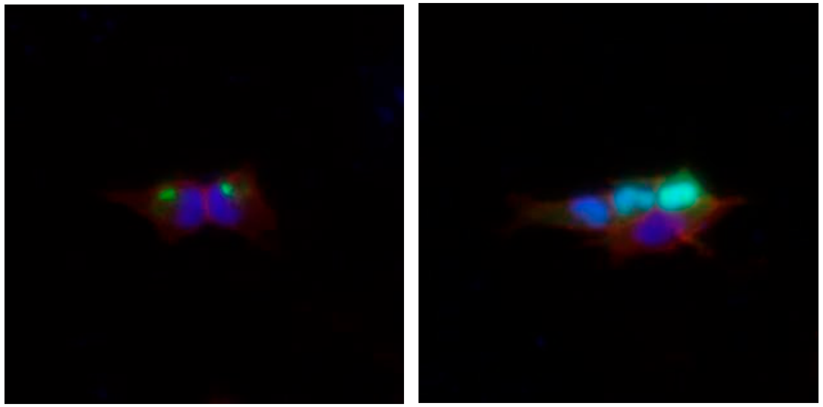

***GRIK2*$^{\text{p.Arg874Gln}}$**　　**GFP**

(C)

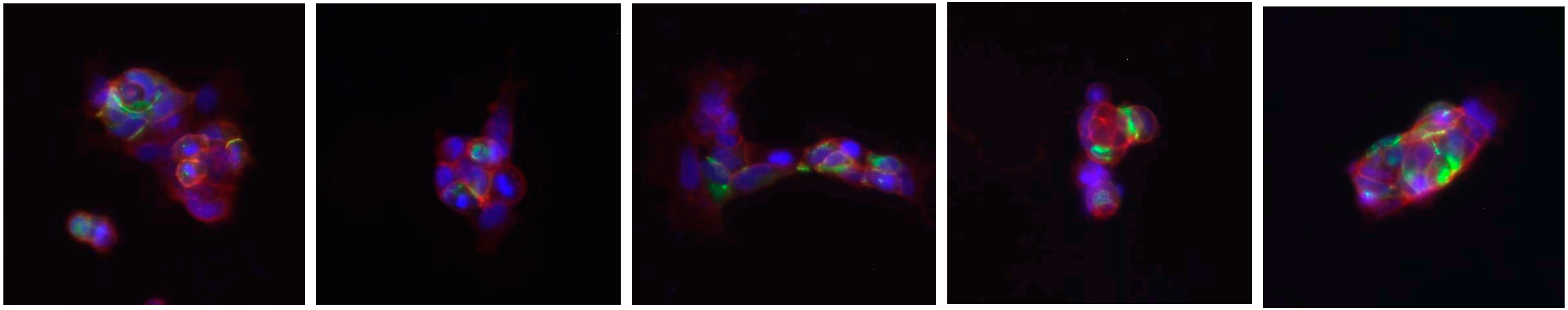

***GRIK3*^{WT}**　　***GRIK3*$^{\text{p.Arg215His}}$**　　***GRIK3*$^{\text{p.Ser310Ala}}$**　　***GRIK3*$^{\text{p.Ser310Pro}}$**　　***GRIK3*$^{\text{p.Gly360Ser}}$**

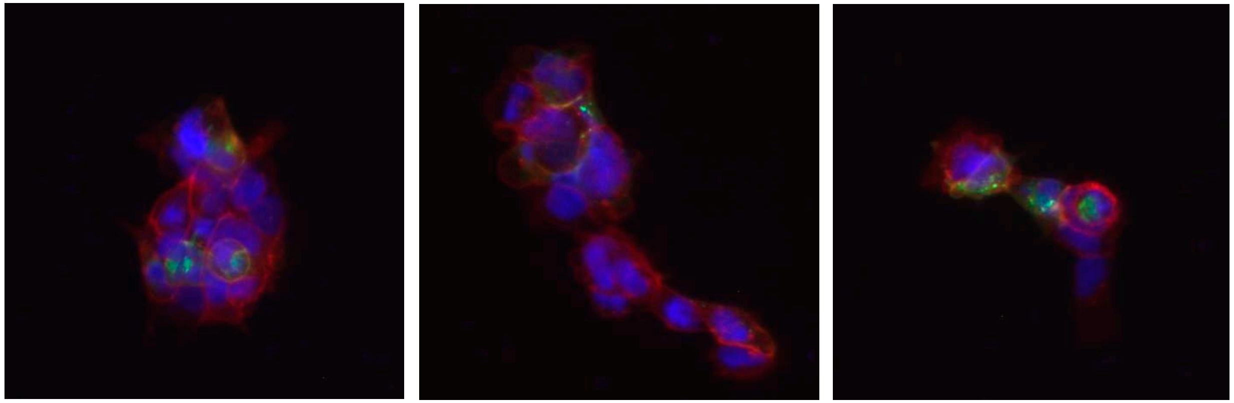

GRIK3$^{\text{p.Val746Ile}}$ *GRIK3*$^{\text{p.Arg867Cys}}$ *GRIK3*$^{\text{p.Pro903Ala}}$

(D)

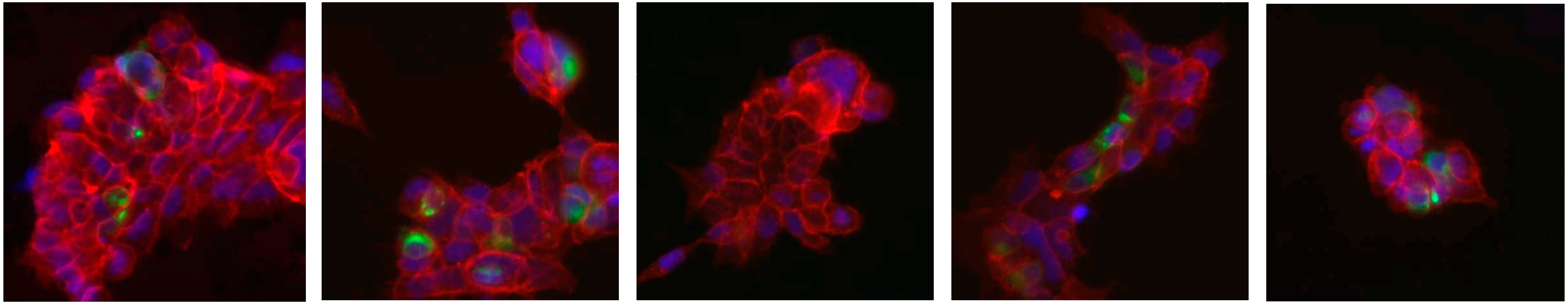

GRIK4^{WT} *GRIK4*$^{\text{p.Ser222Cys}}$ *GRIK4*$^{\text{p.Gln342Ter}}$ *GRIK4*$^{\text{p.Ser402Asn}}$ *GRIK4*$^{\text{p.His403Gln}}$

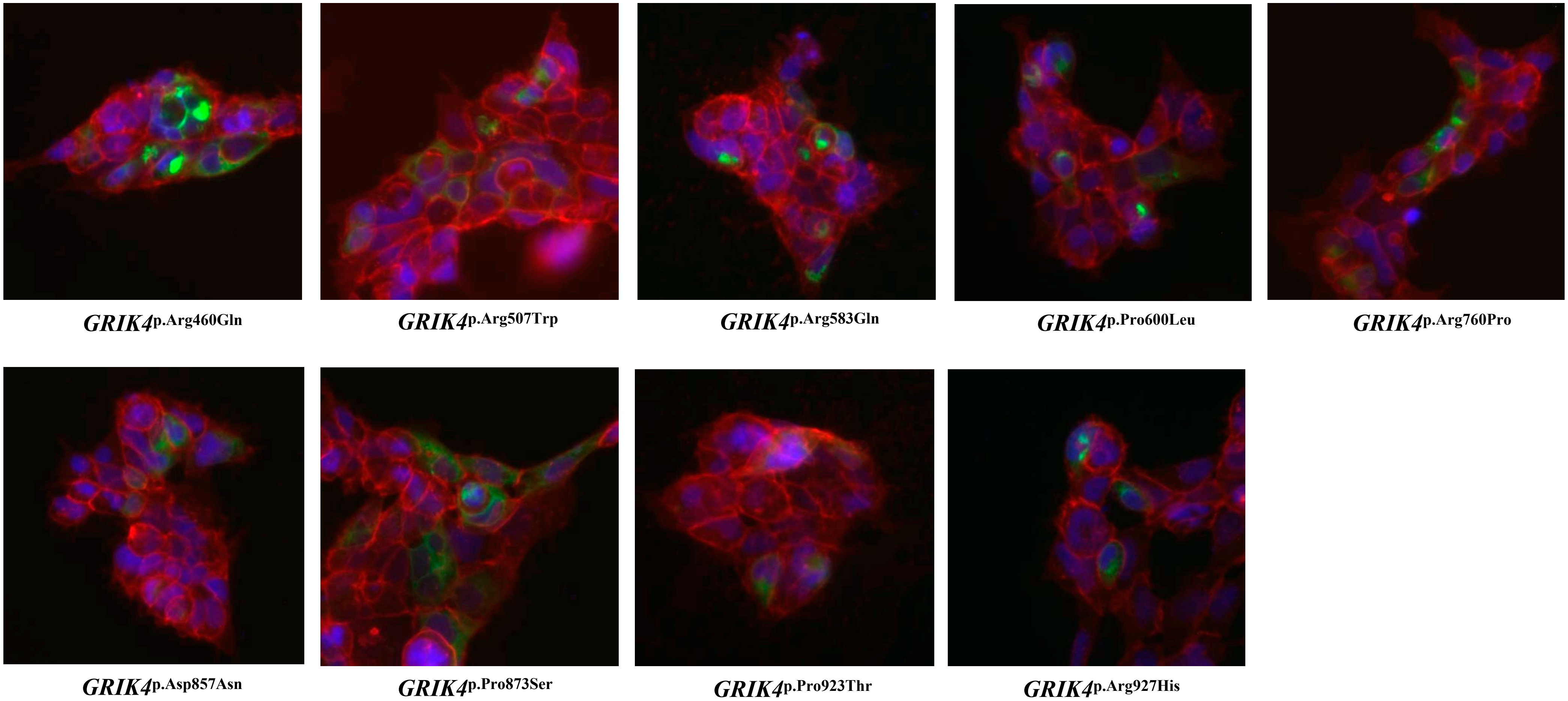
GRIK4 p.Arg460Gln
GRIK4 p.Arg507Trp
GRIK4 p.Arg583Gln
GRIK4 p.Pro600Leu
GRIK4 p.Arg760Pro
GRIK4 p.Asp857Asn
GRIK4 p.Pro873Ser
GRIK4 p.Pro923Thr
GRIK4 p.Arg927His

(E)

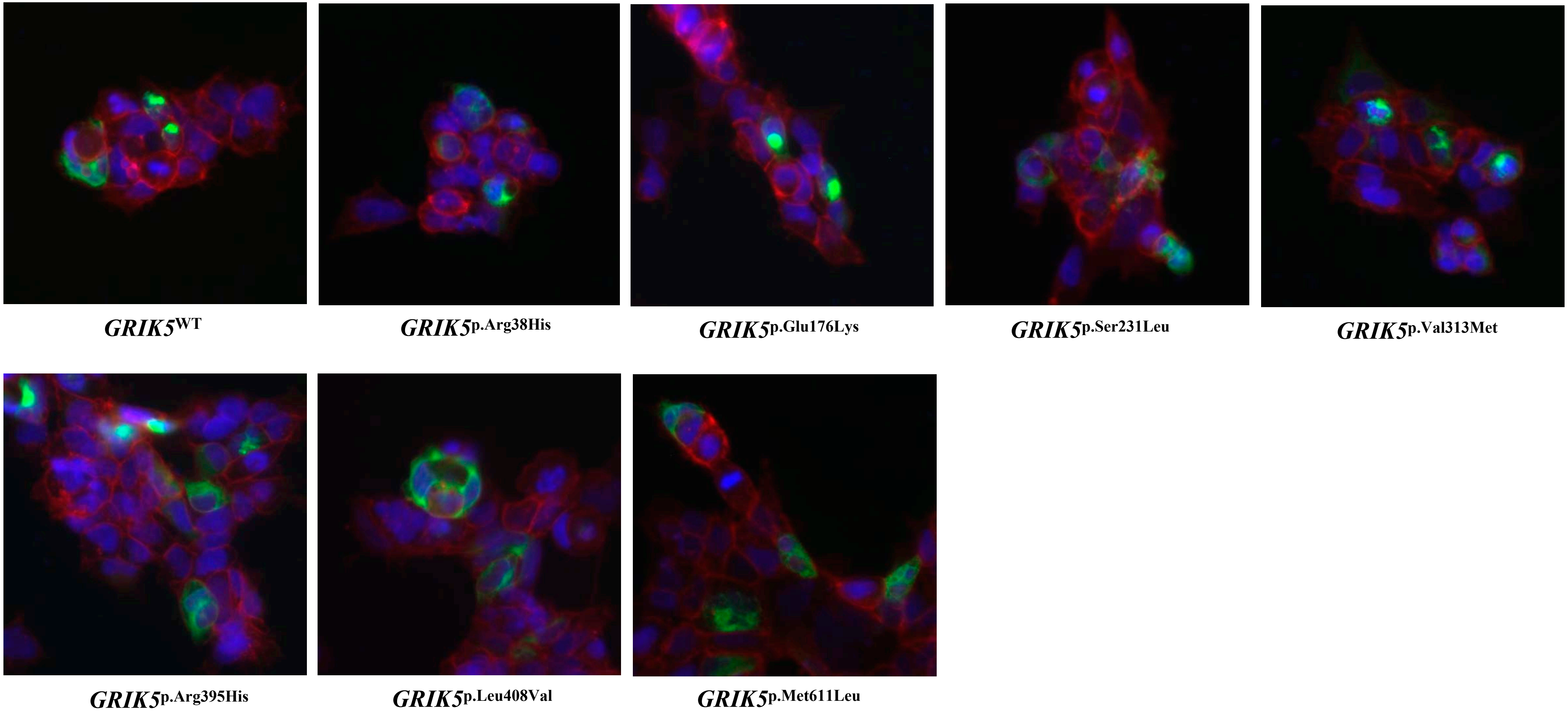

Supplementary Figure 3. Localization analysis of GRIK gene family mutants in HEK-293 cells. (A) GRIK1; (B) GRIK2; (C) GRIK3; (D) GRIK4; (E) GRIK5. Green: target gene. Red: cell membrane marker (wheat germ agglutinin). Blue: cell nuclei (DAPI).
