# Supplementary material for: Ultrarare Loss-of-Function Mutations in the Genes Encoding the Ionotropic Glutamate Receptors of Kainate Subtypes Associated with Schizophrenia Disrupt the Interaction with PSD95
Source: J Pers Med. 2022 May 12;12(5):783. doi: 10.3390/jpm12050783 (PMC9144110; doi:10.3390/jpm12050783)
Supplement: Supplementary file 1 [file jpm-12-00783-s001.zip › Supplementary Table 1 - qPCR primer.pdf]

Supplementary Table 1. Primer sequences of semi-qPCR and size of PCR products.

Amplicon	Target	Forward (5`-3`)	Reverse(5`-3`)	Size (bp)
GRIK1	*GRIK1* expression	ACCCTGATGCCTAACACCAC	AAGAGAGCAGCCACACCAAG	116
GRIK2	*GRIK2* expression	GATTCAGGTTTGCTGGATGG	TCATCTGGGGAAACTGTTGA	100
GRIK3	*GRIK3* expression	CGGAGTCTGGTTTGGGAATA	CGTCCGCATACTCGAAGATT	115
GRIK4	*GRIK4* expression	ATGTCCCACACCATCCTCCT	ATCCACAAGGCTGTCCATTC	114
GRIK5	*GRIK5* expression	TGCCAGGTGCTCTCATCACT	CTCGATGATCCCGTTGATCT	117
GAPDH	*GAPDH* expression	CAATGACCCCTTCATTGACC	GACAAGCTTCCCGTTCTCAG	106
